# Recognizing structure in novel tunes: differences between human and rats

**DOI:** 10.1007/s10071-024-01848-8

**Published:** 2024-03-02

**Authors:** Paola Crespo-Bojorque, Elodie Cauvet, Christophe Pallier, Juan M. Toro

**Affiliations:** 1 Universitat Pompeu Fabra, C. Ramon Trias Fargas, 25-27, CP. 08005 Barcelona, Spain; 2 Cognitive Neuroimaging Unit, INSERM, CEA, CNRS, Université Paris-Saclay, NeuroSpin Center, Gif-Sur-Yvette, France; 3 DIS Study Abroad in Scandinavia, Stockholm, Sweden; 4 Institució Catalana de Recerca I Estudis Avançats (ICREA), Barcelona, Spain

**Keywords:** Music cognition, Rats, Rhythm, Tonality, Familiarization

## Abstract

A central feature in music is the hierarchical organization of its components. Musical pieces are not a simple concatenation of chords, but are characterized by rhythmic and harmonic structures. Here, we explore if sensitivity to music structure might emerge in the absence of any experience with musical stimuli. For this, we tested if rats detect the difference between structured and unstructured musical excerpts and compared their performance with that of humans. Structured melodies were excerpts of Mozart's sonatas. Unstructured melodies were created by the recombination of fragments of different sonatas. We trained listeners (both human participants and Long-Evans rats) with a set of structured and unstructured excerpts, and tested them with completely novel excerpts they had not heard before. After hundreds of training trials, rats were able to tell apart novel structured from unstructured melodies. Human listeners required only a few trials to reach better performance than rats. Interestingly, such performance was increased in humans when tonality changes were included, while it decreased to chance in rats. Our results suggest that, with enough training, rats might learn to discriminate acoustic differences differentiating hierarchical music structures from unstructured excerpts. More importantly, the results point toward species-specific adaptations on how tonality is processed.

**Supplementary Information:**

The online version contains supplementary material available at 10.1007/s10071-024-01848-8.

## Introduction

A key part of humans’ cognitive endowment is the ability to detect hierarchical structures that might be present in signals (Fitch 2014). Such ability is central to the development of higher human capacities such as language (e.g., Morgan and Newport 1981) and music (e.g., Koelsch et al. 2013). Linguistic and musical sequences are not formed by a random organization of elements (words in language and chords in music). Rather, syntactic principles work at different levels in both domains (Patel 2008). For instance, in the construction of words, phrases and sentences in language; and chords, harmonic progressions and keys in music (Patel 2003). However, although there is plenty of research demonstrating that humans efficiently track structure in both language and music, it is yet unknown the extent to which this ability might have its roots in sensitivities already present in non-human animals. In the present study, we tackle this issue by testing whether a distantly related mammal is sensitive to structure in music.

Language and music are produced linearly. We write and speak one word at a time, and we play chords one after other. However, we perceive sentences and tunes in terms of syntactic units. In the sentence “The kid with the cat kicked the ball”, we all understand that the kid kicked the ball, not that it was the cat, even though linearly, the cat is closer to the action than the kid. Thus, syntactic structure is fundamental to how we process language. We tend to repeat the order in which syntactic units are produced in sentences that we have heard recently, a phenomenon known as syntactic priming (Pickering and Branigan 1999). In addition, syntactically incorrect words (e.g., inserting a verb in the position of a noun) readily trigger a signature neural response known as the left-anterior negativity (LAN; Koelsch et al. 2005). Similarly, in music, harmonically incorrect chords (e.g., a dissonant chord that violates a chord progression) consistently trigger an early right-anterior negativity (ERAN) in both highly trained musicians and naïve listeners (Pagès-Portabella and Toro 2020). This evidences that listeners process music and language in terms of syntactic and harmonic hierarchical structures and not just as a succession of local dependencies.

The ability to readily process the complex hierarchical structures defining language and music might well be uniquely human, as other animals such as rhesus monkeys have failed to demonstrate sensitivities to increasingly complex patterns that mirror those present in humans (e.g.,Ferrigno et al. 2020; Jiang et al. 2018). Recent experiments suggest that rodents can track some rhythmic regularities in familiar tunes (e.g.,Celma-Miralles and Toro 2020a, 2020b; Crespo-Bojorque et al. 2022). However, it is possible that humans are the only species able to detect structure in novel tunes, compared to the same tones randomly arranged. We tested this possibility by comparing how humans and rats discriminate between structured and unstructured tunes that they had not heard before.

We presented to listeners a series of melodies with rhythmic and harmonic structure (excerpts of Mozart’s sonatas). We then tested whether they could discriminate novel structured tunes that they had not heard before from tunes composed by the same chords, but completely unstructured (the composing chords were randomized across tunes). In our first experiment, we tested the ability of human listeners to differentiate between the structured and unstructured tunes. In our second experiment, we presented the same stimuli to rats to investigate how the pattern of results observed in humans would compare with those from a species with no musical experience. Our hypothesis is that the detection of harmonic and rhythmic structures is readily available to human listeners even though they have not received formal musical training, although they have been passively exposed to music through their lives. Thus, human participants in our experiment should be able to discriminate the structured from the unstructured musical excerpts. If the ability to detect musical structure needed to discriminate structure from unstructured excerpts is based on general sensitivities already present in other mammals, we should be able to observe similar discrimination capacities in non-human listeners, even if, as the animals in our study, have never been exposed to music before. If, on the contrary, humans’ performance emerges from mechanisms devoted to specific domains (such as music processing; or more generally, to the processing of highly structured stimuli such as language and music), we should observe that non-human animals are unable to discriminate between structured and unstructured musical excerpts. Even more, humans identify musical excerpts as an object that can flexibly vary along surface features such as pitch, tempo and timbre without losing its identity (e.g., Patel 2008). The aim of the present study is to test possible similarities and differences across species in how the structure of music is perceived. Thus, in Experiment 1 and 2 we implemented two conditions. In the single key condition, the tonality of the tunes remained consistent, with all musical excerpts played in the same key, specifically C major. In the multiple keys condition, the tonality of the tunes varied, with each musical excerpt played in a different key.

## Experiment 1

In the present experiment, we explored whether human listeners are able to discriminate between novel excerpts of Mozart sonatas and their unstructured versions. We first trained them to discriminate between a set of structured excerpts from their unstructured counterparts. Then, the participants were tested with completely novel melodies they have not heard during training. Because tonality is a major feature in how we appreciate music, we conducted two conditions. In the single key condition, we tested the listeners’ performance when all the melodies were played using the same tonality (C major). In the multiple keys condition, we tested their performance when the tonality varied across melodies. This allowed us to explore whether they could track the rhythmic and harmonic structures of tunes across changes in tonality.

### Participants

Participants were 32 undergraduate students (23 women; mean age 22 years, 1 month) from Universitat Pompeu Fabra with no formal musical training. They received a monetary compensation for their participation in the study.

### Stimuli

A total of 16 melodies in C major were used to create the stimuli for the single key condition. The melodies were created from excerpts of Mozart’s sonatas and were drawn from the original stimuli used in the doctoral dissertation by Elodie Cauvet (2012). Each excerpt was slightly modified so that it would finish in a perfect cadence (from the fifth to the tonic), which gives a sense of end or resolution to the listener (e.g., Pagès-Portabella and Toro 2020). All stimuli had the same tempo (speed of beat). Each excerpt consisted of sixteen intervals that corresponded to one music bar each (of four beats per bar, known in music terms as 4/4 time). To create the unstructured melodies, the sixteen intervals that composed each of the sixteen original melodies were used. Therefore, we counted on 256 musical intervals from the original melodies, each of which was characterized by the same duration. These 256 intervals were used to create new 16 unstructured melodies of the same duration as the original (structured) ones. Thus, each unstructured melody was the result of an assembly of sixteen intervals, each interval belonging to one of the 16 original melodies. Each interval was used only once for both the structured and unstructured sets. Furthermore, the position of the notes within each melody was respected to create the stimuli. A note that appeared at time 12 in an original melody would appear at time 12 in the unstructured melody. The partitions of the original melodies were transcribed in a sound synthesis software and saved in a Midi format. The beginning and end of each fragment were labeled. A computer program extracted the different fragments of the sound files from the original melodies and recombined them into new Midi files that were converted later to wav format using the software Timidity++ (http://timidity.sourceforge.net/). To explore how structured and unstructured melodies differed from an acoustic point of view, we computed the harmonicity (acoustic periodicity) of each excerpt using Praat (Boersma and Weenink 2023). An independent sample *t* test showed that the mean degree of harmonicity was greater for the structured melodies (*M* = 6.47, SD 2.41) than for the unstructured ones (*M* = 4.75, SD 0.81; *t*(30) = 2.709, *p* = 0.011, 95% CI [0.42, 3.01], *d* = 0.96). This shows that, in fact, scrambling the intervals across melodies to create the unstructured excerpts, increased their acoustic variability.

**Fig. 1:**
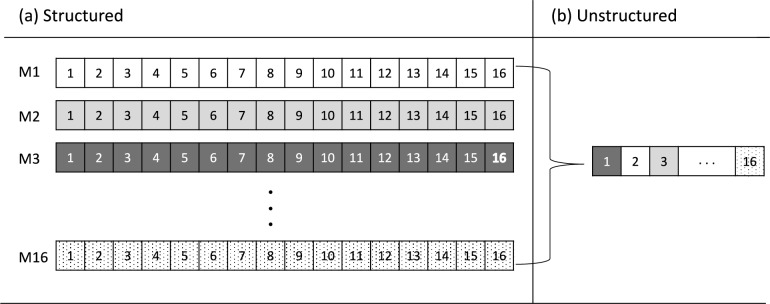
Sixteen structured (**a**) and 16 unstructured (**b**) excerpts used during the experiment. Unstructured excerpts were created by recombining the fragments of different structured melodies. In the single key condition, each melody was played in C major. In the multiple keys condition, each melody was played in a different key

To create the stimuli varying in tonality for the multiple keys condition, the excerpts of the 16 Sonatas were implemented in different major keys: C, C#, D, E♭, E, F, F#, G, A, B, B♭. That is, the excerpts did not all have the same key. As in the previous condition, all excerpts were divided into 16 fragments. To create the 16 unstructured stimuli, we recombined the 256 fragments into 16 new unstructured melodies. These unstructured melodies were thus composed by 16 fragments of different tonalities (Fig. 1).

### Apparatus

Participants were tested individually in a sound attenuating room. Psyscope XB57 software was used to program and run the experiment in a Machintosh OS X-based laptop. The auditory stimuli were presented through Sennheiser HD 515 headphones.

### Procedure

The present experiment consisted of a training phase followed by a test session. Half of the participants (*n* = 16) were presented with stimuli for the single key condition, and half with stimuli for the multiple keys condition. During the training phase we used a go/no-go task. Participants were informed that a randomized set of “correct” and “incorrect" melodies will be presented and that they will have a few seconds to evaluate each melody correctness without any prior guidance or hints on what constituted a correct or incorrect melody. Participants were instructed to press a designated key if they believed a melody to be “correct”, and to refrain from pressing the key if they deemed the melody was “incorrect”. Melodies were presented with an inter-stimulus interval of 3 s independently of participants’ responses. After each response, feedback was displayed on the screen; “correct” for a response after the presentation of a structured melody and “incorrect” for a response after the presentation of an unstructured melody. No feedback was provided if the participant did not respond. A list of ten structured melodies and ten unstructured melodies was randomized and presented at least once during training with the constraint that no more than two stimuli of the same type followed each other. The test phase started once a participant had correctly identified three consecutive melodies as “correct”, with the only restriction that the list of ten structured melodies and ten unstructured melodies was presented at least once (so, each participant had been exposed to at least 20 trials before starting the test phase). If a participant did not meet the criterion of achieving three consecutive correct responses, the training phase extended until this criterion was met or until the list of twenty melodies had been presented twice, allowing for a maximum of 40 trials.

For the test session, we used a two-alternative forced-choice test. Participants were presented with a random list of 12 items, 6 novel structured and 6 novel unstructured melodies not presented during training. Participants were instructed to press a green key on the keyboard if the melody was similar to the “correct” stimuli presented during training or a red key if the melody was similar to the “incorrect” stimuli. We introduced a second key during the test to force the participants to respond in each trial and avoid a conservative strategy of just not responding to any trial (as all the test items were novel for them). Importantly, no feedback was presented during the test session.

### Results

For the training session, we conducted an analysis to determine the number of trials required by participants to grasp the difference between structured and unstructured melodies before proceeding to the test session (see Supplementary Table S1 online). The automated presentation ensured a minimum of twenty trials and a maximum of forty trials.

The mean number of trials needed for participants in the single key condition (*M* = 23.56, SD 6.15) did not differ from those needed for participants in the multiple key condition (*M* = 21.13, SD 2.34; independent sample *t* test *t*(30) = 1.48,* p* = 0.15, 95% confidence interval (CI) [− 0.92 to 5.80], *d* = 0.53).

**Fig. 2:**
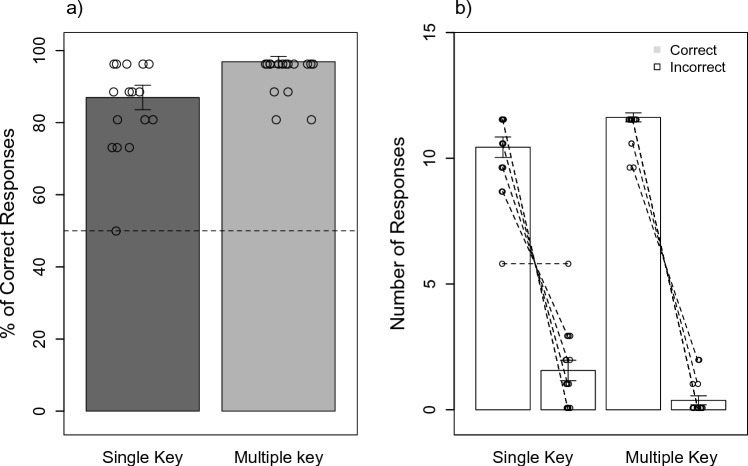
**a** Mean percentage, standard error bars and individual data points (black circles) of human’s correct responses during test. **b** Individual number (single dots) of correct (left bar) and incorrect (right bar) responses during test across the two conditions. Bars show mean and standard error at the group level. Dotted lines connect each individual’s responses. Human listeners discriminated structured from unstructured sequences in both conditions. Playing the melodies in different keys (multiple keys condition) improved their performance

For the test sessions, one-sample *t* test analyses over the percentage of correct responses showed that participants’ performance (percent correct responses) was well above chance (50%) in both conditions: single key condition (*M* = 86.98%, SD 13.60; see Supplementary Table S2 online), *t*(15) = 10.88, *p* < 0.001, 95% confidence interval (CI) [79.73, 94.22], *d* = 2.72); and multiple key condition (*M* = 96.88%, SD 5.99; see Supplementary Table S3 online), *t*(15) = 31.30, *p* < 0.001, 95% confidence interval (CI) [93.68, 100.06], *d* = 7.29). An independent sample *t* test revealed that the performance was significantly better in the Multiple key than in the single key condition (*t*(30) = − 2.66, *p* < 0.05, 95% confidence interval (CI) [− 17.48, − 2.31], *d* = 0.94; see Fig. 2). Thus, even though the listeners were able to systematically recognize structured melodies, changes in tonality made the task easier.

## Experiment 2

In Experiment 1, we observed that human listeners readily discriminated novel structured melodies from their unstructured versions. The key question in the present study is thus, can rats also detect structure in musical excerpts as to tell them apart from excerpts composed by the same tones, but without rhythmic or harmonic structure? The results with human listeners serve as a background against which the performance of rats with no musical exposure can be assessed. We trained rats to discriminate between structured and unstructured melodies and then tested them with novel ones that they have not heard during training.

### Subjects

Animals tested in this study were 32 female Long-Evans rats of 5 months of age. Rats were housed in pairs and were exposed to a 12-h/12h light–dark cycle. Rats had water ad libitum and maintained to 90% of their free-feeding weights. Food was delivered after each training session. Rats can learn to discriminate between sequences of tones in the same range as the ones used in the present study (e.g., Crespo-Bojorque and Toro 2016) and are sensitive to at least some temporal regularities in music (e.g.,Celma-Miralles and Toro 2020a, b; Crespo-Bojorque et al. 2022). In the present study, we used female rats because they tend to produce more responses than male rats. Importantly, however, male and female rats produce the same pattern of responses while processing complex acoustic stimuli (e.g.,de la Mora et al. 2013; Toro et al. 2005), so we expect that the results observed with female rats in the present setting easily generalize to male rats.

### Stimuli

Stimuli were exactly the same as the ones used with humans in Experiment 1. In the single key condition these were structured and unstructured melodies played in C major. In the multiple key condition, structured melodies were played in 11 different tonalities, while unstructured melodies had different keys within them.

### Apparatus

Rats were placed in Letica L830-C response boxes (Panlab S. L., Barcelona, Spain). Each box was equipped with an infrared detector located in the pellet feeder to register nose-poking responses. The stimuli presentation, record of nose pokes, and food delivery were controlled by a custom-made software (RatBoxCBC). A Pioneer A-445 stereo amplifier and two Electro-Voice S-40 loudspeakers (with a response range from 85Hz to 20 kHz), located beside the boxes, were used to present the stimuli at 84 dB.

### Procedure

As in the experiment with the human listeners, the experimental procedure consisted of a training phase followed by a test session. Discrimination training consisted of 50 sessions, one 20-min session per day. Half of the animals (*N* = 16) were assigned to the single key condition and the other half were assigned to the multiple keys condition. In each training session, rats were placed individually in a response box and were presented with 12 structured and 12 unstructured melodies. Each melody was played twice, for a total of 48 melodies presented during each session. The stimuli were played with an inter-stimulus interval of 30 s. The order of presentation of the stimuli was randomized in each session and no more than two stimuli of the same type (structured or unstructured) followed each other. Rats were reinforced for nose-poke responses after the presentation of structured melodies. No food was delivered after the presentation of unstructured melodies independently of nose-poke responses.

After the training phase, a generalization test was run. Similar to a training session, 36 stimuli were presented during test. The only difference was that the 8 test stimuli (4 new structured and 4 new unstructured melodies), repeated twice (16 test items overall) replaced 16 training items. Thus, there were 16 test items interleaved with 20 familiarization items (to avoid response extinction). There was no reinforcement after the presentation of any of the test stimuli. All the experimental procedures were conducted in accordance with the Catalan, Spanish and European guidelines and regulations for the treatment of experimental animals and received the necessary approval from the ethics committee of the Universitat Pompeu Fabra and the Generalitat de Catalunya (protocol number 10557).

### Results

**Fig. 3:**
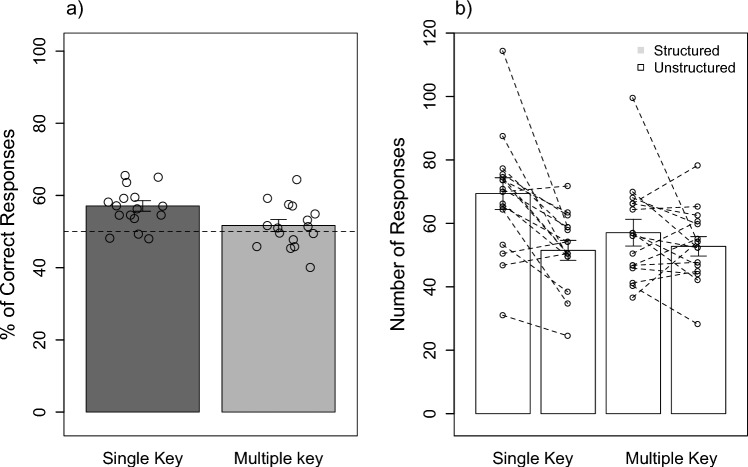
**a** Mean percentage, standard error bars and individual data points (black circles) of rat’s correct responses during test. **b** Individual number of responses (single dots) to the Structured (left bar) and Unstructured (right bar) test stimuli across the two conditions. Bars show mean and standard error at the group level. Dotted lines connect each individual’s responses. Rats discriminated structured from unstructured sequences only in the single key condition. Playing the melodies in different keys (multiple keys condition) dropped rat’s discrimination to chance level

In the single key condition, the animals responded more to the novel structured test items than to the novel unstructured test items (see Supplementary Table S4 online). A one-sample *t* test over the percentage of responses to the structured test items was above what it is expected by chance (*M* = 57.10%, SD 5.84), *t*(15) = 4.87, *p* < 0.001, 95% confidence interval (CI) [53.99, 60.21], *d* = 1.22. Contrary to the single key condition, in the multiple keys condition (see Supplementary Table S5 online), the animals did not respond more to novel structured over unstructured test items (*M* = 51.70%, SD 6.61), *t*(15) = 1.02, *p* = 0.32, 95% confidence interval (CI) [48.17, 55.21], *d* = 0.26. An independent sample *t* test comparing the 2 conditions showed significant differences between them *t*(30) = 2.45, *p* < 0.05, 95% confidence interval (CI) [0.91, 9.92], *d* = 0.87; see Fig. 3. Once changes in tonality were introduced, the animals were not able to discriminate between the structured and unstructured melodies.

## Discussion

Our study points towards similarities and differences in how human and rats discriminate structure in music. When tonality was kept constant, both human and non-human listeners were able to recognize novel melodies that kept a coherent rhythmic and harmonic structure as to discriminate them from their randomized counterparts. However, while human performance was very high after a few learning trials, the rats performed slightly above chance after hundreds of trials distributed along several days of training. The differences across species were even clearer when we changed the key at which the different melodies were played. In humans, the changes in tonality improved their ability to detect unstructured sequences. This contrasts with the results observed with the rats. Once changes in tonality were introduced, the rats were no longer able to discriminate structured from unstructured tunes.

The present results show that, after training, rats might discriminate novel structured from unstructured sequences of chords. However, their performance is much lower than their human counterpart. Humans have a tendency to readily process hierarchical structures, in what has been coined as “dendrophilia” (Fitch 2018), that has not been observed in other animals. In fact, while both human and non-human animals have been observed to learn complex grammars, they display remarkable differences in the speed and accuracy with which they do it (e.g., Jiang et al. 2018). Thus, in the present study, although both human and rat listeners performed above chance in their detection of novel structured sequences, humans clearly outperformed the animals after only a few training trials.

What kind of cues might the listeners in the present study be using to distinguish between structured and unstructured musical excerpts that they have never heard before? By shuffling separate parts of different structured excerpts, unstructured excerpts break the rhythmic and melodic structure of the songs. This is reflected in a significant decrease in harmonicity from the structured to the unstructured excerpts. Both human and rat listeners might thus be able to detect these differences as to tell apart excerpts that they have not heard before. The ability to discriminate regular, isochronous sequences of sounds from irregular ones has been demonstrated in Zebra finches (van der Aa et al. 2015), independently of the tempo at which the sequences are presented (e.g., Rouse et al. 2021) and even in non-vocal learning mammals such as rats (Celma-Miralles and Toro 2020a). Rats can also learn to identify the rhythmic structure of a familiar tune and discriminate it from a version in which such structure is changed (Celma-Miralles and Toro 2020b). It is thus likely that the animals in the present experiment learned to discriminate regular rhythms characteristic of structured excerpts from irregular ones that resulted from the shuffling of different parts of separate songs. But also, both human and rat listeners in the present study might be using harmonic cues to discriminate structured from unstructured songs. Different animal species, including rats (e.g., de la Mora et al. 2013) and Zebra finches (e.g., Spierings et al. 2017), tend to group sequences of sounds following the Iambic–Trochaic law (ITL). The ITL describes how sounds that alternate in intensity and duration are organized in speech and music, with more intense sounds being placed at the beginning of sequences and longer sounds placed at the end (e.g., Bolton 1894). By shuffling separate parts of different songs, unstructured excerpts break down the harmonic alternation of chords that is present in structured excerpts. Thus, a combination of rhythmic and harmonic cues might give enough information to both human and non-human listeners to discriminate structured excerpts from their unstructured counterparts.

Tonal hierarchies (the relations among chords in a melody) are central to how humans perceive music. In fact, it might be one of features universally present across music around the world (Mehr et al. 2019). Even more, both highly trained musicians and naïve listeners readily respond to chords that violate harmonic expectations (e.g.,Koelsch et al. 2000; Pagès-Portabella and Toro 2020). Unsurprisingly, human participants in the present experiment easily discriminated the structured excerpts from the unstructured ones that included changes in tonality and thus broke harmonic cadences. That is, human listeners took advantage of changes in tonality to better discriminate the structured from the unstructured tunes.

In contrast to what we observed with humans, the performance of rats decreased to chance levels in the condition in which we changed the tonality of the excerpts. Evidence regarding the ability of non-human animals to recognize different tones and chord sequences across different tonalities is mixed. While there have been some reports on octave equivalence in rhesus monkeys (Wright et al. 2000) and rats (d’Amato and Salmon 1982), no similar effects have been found in Black-capped chickadees (Hoeschele et al. 2013), budgerigars (Wagner et al. 2019), or European starlings (Cynx 1993). More importantly, Long-Evans rats, as the ones used in the present study, fail to recognize a familiar melody if its pitch is shifted one octave upwards or downwards (Crespo-Bojorque et al. 2022; see also Bregman et al. 2016 for results showing that small alterations in pitch drive melody recognition to chance levels in European Starlings). Such failure is even more surprising as the rats were extensively trained on the discrimination. It also provides a stark contrast with what we observed with human listeners, for whom changes in tonality enhanced discrimination. This suggests that auditory perception in the rats was engaging different processes from those engaged in human listeners.

In the multiple keys condition in our study, structured excerpts were presented at different keys. This might have introduced too much variation across the stimuli for the animals to generalize their common features, such as regular rhythms. Similarly, rats cannot generalize across multiple speakers in a language discrimination task (Toro et al. 2005). On the contrary, if a single speaker is used, they can effectively perform the target discrimination (Toro et al. 2003). This suggests that acoustic variability, such as the one introduced in our study by playing the excerpts at different tonalities, masks common features that the animals might use to discriminate structured from unstructured sequences. It would be interesting, however, to test other species with a similar task. For example, it is an open question whether songbirds, who have extensive experience producing and processing complex vocalizations would display an increased performance in both the single key and multiple keys conditions. Similarly, it would be interesting to explore whether animals with ample experience with multiple human speakers, such as pet dogs, could in fact benefit from the multiple keys condition as we observed with human listeners in the present study.

Complementarily, the human participants in our study were presented with the test once they reached a training criterion (identification of three consecutive melodies as “correct”), which they did very rapidly, usually within the first 20 trials. In contrast, there was no training criterion that the rats had to reach to move to the test phase. They all were presented with stimuli during 50 training sessions. It thus might be the case that if the test was presented to the rats only after they had reached a given learning criterion, their performance might have increased. With the present results, however, we observe that humans’ performance after only 20 training trials is consistently better than rats’ performance after more than 2 thousand trials.

The methods used in the present study are inspired by research on the processing of linguistic structure. Exploring the cerebral mechanisms underlying the processing of hierarchical structures, Pallier et al. (2011) developed a method to compare the responses to syntactically coherent sequences and those to acoustically similar sequences that increasingly lacked syntactic coherence. In their experiment, they used 12-word sentences that were composed by one syntactic constituent (e.g., “I believe that you should accept the proposal of your new associate”). Across different conditions, they divided the sentences into smaller segments that were mixed across sentences. With this procedure, Pallier and collaborators were able to create acoustically similar sequences while degrading syntactic structure. Thus, the authors contrasted the original sentences with sentences in which the 12-word streams were composed by more constituents (e.g., “mayor of the city he hates this color they read their names”; that is, a sentence composed by 3 4-word constituents). The most extreme contrasting condition involved a stream of 12 words with no syntactic relation between them (e.g., “thing very tee where of watching copy tensed they states heart plus”). The logic behind this design is that the human brain should react differently to structured streams when compared with streams composed by the same number of words but with no structure. In fact, the authors demonstrated that left-hemisphere regions showed increased activation that correlated with the increased number of syntactic constituents present in a sentence.

Shuffling segments of syntactically coherent sentences to create stimuli that preserves acoustic features at the local level but that lacks syntactic coherence is a method that has been successfully used in other studies. Overath et al. (2015) used what they called “quilts” to investigate the brain responses to speech structure while eliminating lexical, syntactic or semantic processes. The authors divided sentences into segments that were reordered and put together. This created sequences that kept speech features, but that lacked syntactic or semantic coherence (a similar manipulation has been recently used to create control stimuli in a language discrimination study with dogs; see Cuaya et al. 2022). More important for the present study, these manipulations can also be implemented over music segments. This creates novel sequences that lack the temporal structure of music (e.g., Abrams et al. 2011). It thus offers an opportunity to compare how listeners react to syntactically correct sequences of chords with sequences composed by the same chords, but with no harmonic or rhythmic structure. We took advantage of this manipulation to compare across species (humans and rats) the abilities to discriminate structured from unstructured musical excerpts.

Comparative research has shown that basic abilities involved in language and music processing might be the result of general acoustic biases not specific to humans (e.g.,Hoeschele et al. 2015; Patel 2019). That is, the abilities required for the emergence of language and music might arise from pre-existing sensitivities that evolved in other species. Whether hierarchical processing is one of these pre-existing abilities is a critical question to explore the evolutionary origins of human cognitive skills. The present results contribute to this line of research by demonstrating some commonalities but also by highlighting important differences in music perception in humans and rats. After hundreds of training trials, the rats were able to discriminate between completely novel tunes that they have not heard before. They might do so by focusing on regular rhythms and chord alternations. In contrast, human listeners readily discriminated those tunes after a few trials. More importantly, while human listeners took advantage of changes in tonality across music excerpts, the performance in rats is impaired by the increase in acoustic variance that such changes involve.

## Supplementary Information

Below is the link to the electronic supplementary material.

Supplementary file1 (DOCX 28 kb)

## Data Availability

All primary data are included in the Supplementary material. All stimuli are uploaded in the Open Science Framework (https://osf.io/a4m6r/?view_only=889611d3933f4fa4be5d967b273a3e98).
