# Supplementary material for: Recognizing structure in novel tunes: differences between human and rats
Source: Anim Cogn. 2024 Mar 2;27(1):17. doi: 10.1007/s10071-024-01848-8 (PMC10907461; doi:10.1007/s10071-024-01848-8)
Supplement: Supplementary file 1 — Supplementary file1 (DOCX 28 kb) [file 10071_2024_1848_MOESM1_ESM.docx]

**Supplementary Material**

**Recognizing structure in novel tunes: Differences between human and rats**

Paola Crespo-Bojorque, Elodie Clauvet, Christophe Pallier, Juan M. Toro

**S1 *Supplementary Table S1*. Human listeners – training.**

| **Single key** | | **Multiple keys** | |
| --- | --- | --- | --- |
| **Participant** | **Trials** | **Participant** | **Trials** |
| **1** | 20 | **17** | 20 |
| **2** | 34 | **18** | 23 |
| **3** | 23 | **19** | 20 |
| **4** | 20 | **20** | 20 |
| **5** | 32 | **21** | 20 |
| **6** | 23 | **22** | 20 |
| **7** | 20 | **23** | 20 |
| **8** | 22 | **24** | 20 |
| **9** | 20 | **25** | 22 |
| **10** | 20 | **26** | 20 |
| **11** | 20 | **27** | 20 |
| **12** | 23 | **28** | 28 |
| **13** | 20 | **29** | 20 |
| **14** | 40 | **30** | 25 |
| **15** | 20 | **31** | 20 |
| **16** | 20 | **32** | 20 |

*Note.* Number of trials presented during training for each participant and condition.

**S2 *Supplementary Table S2*. Human listeners – Single key.**

| **Single key condition** | | | |
| --- | --- | --- | --- |
| **Participant** | **Correct** | **Incorrect** | **% Correct responses** |
| **1** | 11 | 1 | 91.67 |
| **2** | 10 | 2 | 83.33 |
| **3** | 9 | 3 | 75 |
| **4** | 11 | 1 | 91.67 |
| **5** | 10 | 2 | 83.33 |
| **6** | 6 | 6 | 50 |
| **7** | 12 | 0 | 100 |
| **8** | 9 | 3 | 75 |
| **9** | 11 | 1 | 91.67 |
| **10** | 12 | 0 | 100 |
| **11** | 9 | 3 | 75 |
| **12** | 12 | 0 | 100 |
| **13** | 11 | 1 | 91.67 |
| **14** | 12 | 0 | 100 |
| **15** | 12 | 0 | 100 |
| **16** | 10 | 2 | 83.33 |

*Note.* Number of correct and incorrect responses during test along with the corresponding percentage of correct of responses for each participant.

**S3 *Supplementary Table S3*. Human listeners – Multiple keys.**

| **Multiple keys condition** | | | |
| --- | --- | --- | --- |
| **Participant** | **Correct** | **Incorrect** | **% Correct responses** |
| **17** | 12 | 0 | 100 |
| **18** | 11 | 1 | 91.67 |
| **19** | 11 | 1 | 91.67 |
| **20** | 12 | 0 | 100 |
| **21** | 12 | 0 | 100 |
| **22** | 12 | 0 | 100 |
| **23** | 12 | 0 | 100 |
| **24** | 12 | 0 | 100 |
| **25** | 10 | 2 | 83.33 |
| **26** | 12 | 0 | 100 |
| **27** | 12 | 0 | 100 |
| **28** | 10 | 2 | 83.33 |
| **29** | 12 | 0 | 100 |
| **30** | 12 | 0 | 100 |
| **31** | 12 | 0 | 100 |
| **32** | 12 | 0 | 100 |

*Note.* Number of correct and incorrect responses to during test along with corresponding percentage of correct of responses for each participant.

**S4 *Supplementary Table S4*. Rats – Single key.**

| **Single key condition** | | | |
| --- | --- | --- | --- |
| **Rat** | **Structured** | **Unstructured** | **% Correct responses**  **(structured)** |
| **1** | 66 | 54 | 55 |
| **2** | 46 | 50 | 47.92 |
| **3** | 65 | 33 | 66.33 |
| **4** | 119 | 59 | 66.85 |
| **5** | 76 | 50 | 60.32 |
| **6** | 77 | 63 | 55 |
| **7** | 67 | 49 | 57.76 |
| **8** | 53 | 37 | 58.89 |
| **9** | 29 | 22 | 56.86 |
| **10** | 75 | 50 | 60 |
| **11** | 75 | 64 | 53.96 |
| **12** | 50 | 54 | 48.08 |
| **13** | 72 | 59 | 54.96 |
| **14** | 90 | 49 | 64.75 |
| **15** | 71 | 73 | 49.31 |
| **16** | 79 | 58 | 57.66 |

*Note.* Number of nose-poking responses to structured and unstructured melodies during test along with the corresponding percentage of correct responses for each animal.

**S5 *Supplementary Table S5*. Rats – test.**

| **Multiple keys condition** | | | |
| --- | --- | --- | --- |
| **Rat** | **Structured** | **Unstructured** | **% Correct responses**  **(structured)** |
| **17** | 50 | 61 | 45.05 |
| **18** | 67 | 80 | 45.58 |
| **19** | 56 | 52 | 51.85 |
| **20** | 71 | 52 | 57.72 |
| **21** | 65 | 66 | 49.62 |
| **22** | 57 | 46 | 55.34 |
| **23** | 103 | 54 | 65.61 |
| **24** | 46 | 47 | 49.46 |
| **25** | 35 | 54 | 39.33 |
| **26** | 69 | 60 | 53.49 |
| **27** | 45 | 43 | 51.14 |
| **28** | 40 | 44 | 47.62 |
| **29** | 39 | 26 | 60 |
| **30** | 46 | 55 | 45.54 |
| **31** | 67 | 63 | 51.54 |
| **32** | 57 | 41 | 58.16 |

*Note.* Number of nose-poking responses to structured and unstructured melodies during test along with the corresponding percentage of correct responses for each animal.
